# P05-03 The role of physical training in the correction of cognitive impairment in patients with type 2 diabetes

**DOI:** 10.1093/eurpub/ckac095.070

**Published:** 2022-08-29

**Authors:** Mariia Matveeva, Julia Samoilova, Natalie Zhukova, Inessa Yakimovich

**Affiliations:** 1 Endocrinology and diabetology, Siberian State Medical University, Tomsk, Russia; 2 Neurology and neurosurgery, Siberian State Medical University, Tomsk, Russia; 3 Hyguene,Siberian State Medical University, Tomsk, Russia

**Keywords:** physical training, cognitive impairment, type 2 diabetes, quality of life

## Abstract

**Background:**

The purpose of the study was to evaluate the role of physical exercises in improving cognitive functions in type 2 diabetes mellitus.

**Methods:**

The study protocol was approved by an ethics committee and all patients signed an informed consent. We examined 204 patients with type 2 diabetes aged 61.7 ± 11.2 years (persons hospitalized in the endocrinology department of the clinics of the Siberian State Medical University, Tomsk). Blind double method patients were randomized into 2 groups: the main one was engaged in physical therapy and the control group (observation). The study was carried out in two stages: at the first visit, a clinical and psychological examination was conducted, which included the Montreal Cognitive Function Assessment Scale and the Diabetes-dependent Quality of Life Questionnaire; repeated clinical and psychological examination was carried out after rehabilitation after 6 months

**Results:**

At the first stage the study revealed the presence of cognitive impairment in type 2 diabetes, mainly in tasks on visual-constructive skills, memory, attention and speech. These disorders were reduced after physical therapy (second stage) by 2.6 points (t = 0.01, p = 0.00006): visual-constructive skills (t = 0.0, p = 0.008), speech (t = 0.0, p = 0.005), abstraction (t = 0.0, p = 0.002) and memory (t = 0.0, p = 0.0007), no change in cognitive function occurred in the observation group. In terms of carbohydrate metabolism: the level of HbA1c decreased by 0.9%, fasting glycemia - by 1.6 mmol/L in main group, but increased by 0,1% of HbA1c and by 0,2 mmol/L of glycemia in the observation group (second stage) (t = 2.0, p = 0.000003; t = 3.0, p = 0.0008). Patients involved in physical therapy showed improvement in a larger number of parameters - leisure, travel, vacation, personal life, appearance, self-confidence, future confidence, financial situation, dependence, food choice, choice of drinks, total point (p > 0.05), when there was a decrease in points in the parameters of the control group.

**Conclusion:**

During rehabilitation in patients with type 2 diabetes, there is an improvement in cognitive functions, carbohydrate metabolism and quality of life.

